# A New Plan on Medical Reform

**Published:** 1847-03

**Authors:** 


*A New Plan on Medical Reform*—To the Editor of the Boston Medical and Surgical Journal—My Dear Sir,—The causes that contribute to the origin and sustenance of empiricism, are subjects of interesting investigation at the present time. I think it a matter of regret, that many influential persons, fired by an ill-judged scientific zeal, have endeavored, by sober argumentation and rules of logic, to demolish the prevailing systems of quackery. All past experience proves, very conclusively, that to convince a man's judgment when his prejudices are enlisted on the opposite side, is a hopeless undertaking. Who ever heard of a single convert being made by a religious controversy? The antagonists themselves commence their set-to in all the over-boiling exuberance of christian charity—like a couple of friends sparring. One finally gives the other a dab which sets his nose to bleeding; he retaliates, and their light sparring becomes a serious matter of fist and scull.

Our friends of the *schools militant* commence their attack upon the quacks, by the declaration of sundry sound and indubitable aphorisms—such as "truths are stubborn things," &c. To this I reply, "and so are asses;" there is nothing more difficult than to drive one of these long-eared gentry one way, when he pertinaciously sets his mind upon travelling another. Send a country lad to drive a pig: does he endeavor by compulsion to get the contumacious brute to walk off in the desired direction? Not he; he knows by experience that he would only get his labor for his pains—the pig, like Falstaff, will "give no man a reason on compulsion." The only way to succeed easily is to make the spirit of insubordination subserve his purposes, and he catches the animal by the tail to pull him in the opposite direction. Any other plan, he will tell you is all gammon.

The wrong plan has been adopted for the opposition of homœopathy. Denunciations have been forged, and hurled with thundering sound, to no effect, and the credulity which enshrouds men's faculties, leaves them blind and willing victims to the doctrine of infinitesimal doses. The system has been handled with rough ceremony, and the monstrous faith is less-than-nothing doses assailed with the fury and indignation so easily excited by a threatened invasion of pecuniary interest; but the gaping crowd still swallow the little powders, and Herr Homœopath laughs in his sleeve as he pockets the fat fees so easily fished from the pockets of credulous hypochondriacs and hysterical women.

You are wrong, gentlemen! Cease your opposition; admit the truth of Hahnemann's nonsense; nay, outstrip him in fertility of invention and deception. If a homœopath tells you that a globule of sugar, moistened with the 30th dilution of a given remedy, and applied to the nostrils of a patient in extremis, will relieve him, reply to him, and shout to the world that we have a remedy, so exquisitely powerful in its influence upon the animal machine, and only known to allopathic physicians, that the same globule moistened with the 300 dilution (!!!) and applied to the nether end of a dead man, will bring him to life! You must learn the game of brag, and always "go better." Try your d—st (excuse Kentucky vernacular) to persuade people that there is really nothing strange in homœopathy, compared with some half-hatched system with which you are about to astound the world, catch the pig by the tail, and two to one the "Duch doctors" will soon be found upon some other hobby, denouncing their quondam favorite as the most insignificant, irrational, and transparent hoax that was ever devised and attempted.

So with hydropathy. If Priessnitz swears that he cures his patients by pouring cold water by the gallon down their throats, turn up your noses at him, and tell the world that you are much more successful by squirting buckets-full of hot water up the back way.*

He assails the enemy in front, you behind—he carries the citadel by storm, you by surprise; and I appeal to all authority to decide which manœuvre is the safest and best. If he publishes tables of cases that show a success amounting to 75 per cent., you publish larger tables, and claim 95 per cent.! Admitting thatyou do not adhere to veracity, and that you are charged with it; you may be thankful that it is so, raise the cry of persecution, and your fortunes are sure.

A good while since, after Harvey had enlightened us concerning the circulation, it was announced to the world that life might be preserved ,*ad infinitum*, by the process of transfusion. Old people pricked up their ears, and eagerly stretched out their emaciated arms to receive anew the vital current from a sheep! What a captivating idea! The grand secret of earthly immortality resting upon the piston of a pewter squirt! How the sublime blends down into beautiful harmony with the ridiculous! For a time syringes "looked up." But it was soon discovered that this great idea was "as the baseless fabric of a vision." And yet this was the wisdom of Solomon, compared with some notions fashionable in our day of new lights.

Homœopathy is certainly a very popular delusion, and, like some other delusions, exceedingly agreeable, if we could only persuade ourselves of its truth. Who would not rather be cured, "*cito et juncunde*," by the sugar of milk, than to die, "*secundem artem*," under the remorseless fire of a "regular practitioner's" prescription? What if a man is told, by sneering opponents of the system, that the homœopathic medicine is a very near approach to pap, and that it is exceedingly appropriate to his infantile credulity!

Let those laugh that win. There has been a good deal of speculation concerning the origin of homœopathy. It has been attributed

---

* The sobriety of grave professional reading, needs the occasional relaxation of mirthfulness. The sprightly humor of this communication will perhaps divert our readers; but we cannot say that it is calculated "to point a moral or adorn a *tail*.

BUFFALO MEDICAL JOURNAL.

to ignorance, superstition and craft, and some are even uncharitable enough to believe that Hahnemann himself acknowledged, before his death, that it was all a humbug. I profess, Mr. Editor, to be an observing man, and I think I can explain the matter to the satisfaction of every reasonable individual, of course includiug yourself in the category.

You remember, doubtless, that in old times people had no nerves —the old gentleman in the play said that he never had any in his life. Nerves and hysterics are things of purely modern invention, The "vapors", and the "blues" owe their existence to the "conventionalities of fashionable society." The hyper-sensibility which has, in these latter days, come to be considered the indispensable of refinement and fashion, seems to have extended to the stomach and bowels. A while since, an honest, rousing dose of physic was required to make an impression upon the sturdy organs of a patient—the encounter between the doctor and the disease was a fair stand-up fight, soon ended with hard blows, and no favors asked. But the fashion of us moderns, which makes a man the creation of starched dickies, high-heeled boots and tight waistcoats— the thing of a barber's brush and the tailor's yard stick ; and angelic woman, a swaddling lusus—a heterogeneous compound of wad of cotton, French chalk, buckram, and strips of whalebone, has drawn so exquisitely fine the delicate cords of sensibility, that the "30th dilution" applied to the nose proves perfectly overpowering. There are thousands of persons, now-a-days, of both sexes, who under proper circumstances, can die Pope's aromatic death.—Of course they come to life again, modestly expecting the performance to be encored! Great heaven! What is the world coming to, when sacred sensibility is worn as a harlequin's dress, to amuse an audience, and monkeys are become the highest objects of emulation to mankind? "Just to that point [remarks an ill-natured friend at my elbow] which so far divests them of common sense, as to make men credulous of infinitesimal agencies." Softly, My dear sir, we must take the world as we find it.

Do you not perceive that Hahnemann's system is the offspring of necessity and of nerves? You would begin your reformation where it ought to end: if you restore mankind to a state of health, bodily and mentally, and blunt by proper education the morbid sensibility of the nerves, homœopathy will die a natural death ; but destroy at once the little globules, and what becomes of human nature!

Besides all this, Mr. Editor, we profess to be a little wiser than our fathers. I fancy, sir, that we require something a little more pretending than sheep saffron and barn-yard poultices to suit the taste of the present generation. If we cure disease by conjuration which they encountered with the awful list of pills, portionsand-plasters ; why not? We can even quote precedent for our practi ces. There was a famous pill, celebrated in Pindaric verse, which, with your permission, I will copy.

" A bumpkin came among the rest,
And thus the man of pill addressed:
'Zur, hearing what is come to pass,
That your fine pill hath cured the king,
And able to do everything,
D'ye you think, zur, t'will make me find my ass?
I've lost my ass, zur, zo should like to try it;
If this be your opinion, zur, I'll buy it.'
'Undoubtedly!' the quack replied,
'Yes, master Hob, it should be tried.'
Then down Hob's gullet, cure or kill,
The grand imposter pushed the pill,
Hob paid his fee, and off he went;
And traveling on about an hour,
His bowels sore with pains were rent;
Such was the pill's surprising power,
No longer able to contain,
Hob in a hurry left the lane,
And sought the grove—where Hob's two eyes,
Wide staring, saw with huge surprise
His long-eared servant Jack, his ass!!
'Adzooks! a lucky pill!' quoth Hob;
'Yes, yes, the pill hath done the job.'

"Globules (remarks again my crusty friend) have discovered more asses in these times than did Pindar's pills; and what is stranger, all are affected with the mange, the itch, or—something worse!" But, my good sir, this is not the fault of the system of Hahnemann. That fact does not condemn, by any means, the sugar of milk; only the mal-practice, and filthy habits of the times. We must do penance, in mercury and sulphur, for past peccadilloes, and thank God if this is the nearest acquaintance we are destined to have with brimstone. Alopathy has done nothing more (we are told,) in 2500 years, than to discover these two specifics, and homœopathy, forsooth, must teach her to employ these properly! It remains to be seen what the "Young Physic," recently born under Dr. Forbes' obstetric management, will accomplish.—Uutil then, with an apology for the length of this straggling epistle, allow me to subscribe myself, with great respect,

Your ob't servant,

*Lexington, Ky., Nov. 14th,* 1846. OLD PHYSIC.

